# Are the SORG and OPTImodel, Tokuhashi and Tomita Algorithms Still Suitable as Predictors of Survival in Patients With Vertebral Metastases in Routine Clinical Practice?

**DOI:** 10.1002/cam4.71520

**Published:** 2026-02-03

**Authors:** Julián Cabria Fernández, Pablo González‐Herráez Fernández, Javier Mateo Negreira, Pedro Arcos González

**Affiliations:** ^1^ Department of Orthopedic Surgery and Traumatology Cabueñes University Hospital Gijón Spain; ^2^ Department of Medicine University of Oviedo Oviedo Spain

## Abstract

**Objectives:**

To evaluate the performance of the Tokuhashi, Tomita, SORG machine learning (SORG ML), and OPTImodel algorithms as survival predictors for vertebral metastases in clinical practice.

**Materials and Methods:**

A retrospective study (2013–2023) analyzed 573 patients from Cabueñes University Hospital (Asturias, Spain). Thirty‐two demographic, epidemiological, clinical, and analytical variables were considered, including diagnosis chronology and survival.

**Results:**

Among the 573 patients studied, 272 (47.4%) presented visceral metastases at the time of diagnosis. A total of 362 patients (63.2%) had associated comorbidities. The most frequent primary histological diagnoses in these patients were lung 147 (25.7%), prostate 146 (25.5%), breast 118 (20.6%), kidney 30 (5.2%), and colorectal 29 (5.1%). The median survival of the cohort was 185 days. The accuracy rates for the Tokuhashi, SORG ML, OPTImodel, and Tomita algorithms were 0.5509, 0.4812, 0.3404, and 0.3858, respectively. The models with the highest accuracy rates in specific time segments were Tokuhashi (77.5% for < 6 months) and OPTImodel (90.8% for more than 1 year). The areas under the curve (AUC) for survival intervals were as follows: Tokuhashi at 42 days (73.19%), 90 days (79.3%), and 365 days (82.73%); Tomita at 42 days (69.27%), 90 days (76.82%), and 365 days (78.79%); SORG ML at 42 days (52.77%), 90 days (51.69%), and 365 days (51.38%).

**Conclusions:**

All models showed relatively low accuracy. The newer models (OPTImodel, SORG ML) did not outperform the traditional Tomita and Tokuhashi in predicting survival for vertebral metastases patients.

## Graphical abstract

The study evaluated survival prediction algorithms for vertebral metastases, comparing Tokuhashi, Tomita, SORG ML, and OPTImodel. Tokuhashi showed the highest accuracy for < 6 months (77.5%) and OPTImodel for > 1 year (90.8%). Overall, newer models (SORG ML, OPTImodel) did not outperform classic methods, highlighting limitations in predicting survival in clinical practice.

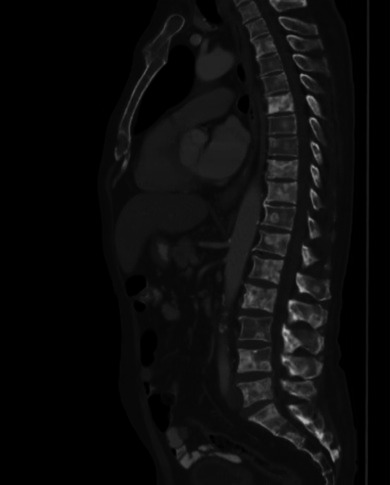

## 1 Introduction

Bone metastases occur in the most advanced stage of tumor disease, and their most frequent location is the vertebrae, often being multiple at the time of diagnosis [1]. Metastases account for up to 90% of all malignant bone tumors in adulthood [2, 3], and their incidence is increasing due to several factors, including improved survival of patients receiving targeted oncological therapies and enhanced diagnostic accuracy with more precise imaging tools [4].

The disease is typically diagnosed in its advanced stages, and treatment is predominantly palliative. Surgical treatment with curative intent is only feasible in a limited number of cases. Therefore, there is consensus that a minimum survival of 3 months is necessary for the temporary loss of quality of life caused by extensive surgery to be clinically justified for the patient [5]. It is important to note that surgical management includes not only extensive or curative procedures but also palliative interventions such as decompression, stabilization, or analgesic surgery when other therapeutic options have failed.

Proper selection of candidates is crucial, and various prognostic algorithms have been developed based on clinical, analytical, and histological variables. Unfortunately, there is currently no clear consensus on the superiority of one algorithm over others [6].

Among the most commonly used algorithms are those developed by Tokuhashi in 1990 [7] and revised in 2005 [8], by Tomita in 2001 [9], by Bauer in 1995 [10] and modified in 2008 [11], by van der Linden in 2005 [12], by Katagiri in 2014 [13], by Ghori in 2015 [14], the OPTImodel [15] and NESMS [16] models (both from 2016), and the SORG algorithm from 2016 [17], along with its updated version in nomogram form from 2017 [18] and its 2019 machine learning‐based version (SORG ML) [19].

The objective of this study is to evaluate the current performance of the Tokuhashi, Tomita, SORG ML, and OPTImodel algorithms as survival predictors in patients with vertebral metastases in routine clinical practice.

## 2 Materials and Methods

A retrospective observational study was conducted on 622 patients diagnosed with vertebral metastases at Cabueñes University Hospital (Gijón, Spain) between 2013 and 2023. Inclusion criteria were as follows: patients had to be over 18 years old at the time of diagnosis, have an imaging test (CT, MRI, bone scintigraphy, etc.) confirming the location of the metastases, and have a complete record of all variables collected in their clinical history.

The studied variables included: sex, age at diagnosis, number of bone metastases, number of vertebral metastases, presence or absence of visceral metastases, feasibility of treating these metastases with any of the three standard treatment modalities (chemotherapy or targeted therapy, radiotherapy, or surgery), presence or absence of lymphatic metastases, presence or absence of brain metastases, presence or absence of pathological fractures, functional status using the ECOG and Karnofsky scales, degree of neurological impairment based on the ASIA scale, primary tumor diagnosis, presence or absence of comorbidities according to the Charlson scale, use of prior systemic therapy, and body mass index (BMI).

Analytical variables recorded included: hemoglobin (g/dL), platelets (×10^3^/μL), leukocytes (×10^3^/μL), lymphocytes (×10^3^/μL), neutrophils (×10^3^/μL), serum creatinine (mg/dL), international normalized ratio (INR) of prothrombin time, alkaline phosphatase (IU/L), and serum albumin (g/L). Additionally, the vital status of each patient (alive or deceased) at the study's end date (February 2024) was compared with the survival estimates provided by the Tokuhashi, Tomita, OPTImodel, and SORG ML predictive models.

When applying the Tokuhashi, Tomita, SORG ML, and OPTImodel algorithms, estimated survival scores at 90, 180, and 365 days were obtained, respectively. For the SORG ML algorithm, the estimated survival percentages for these intervals were directly provided by its online tool [20].

For the distribution analysis of the variables, central tendency parameters such as mean, median, standard deviation (SD), and 95% confidence intervals (95% CI) were used. Sensitivity, specificity, and positive and negative predictive values of each algorithm were calculated based on confusion matrices. Receiver operating characteristic (ROC) curves were used to establish the corresponding area under the curve (AUC), and the Kolmogorov–Smirnov test was used to assess the normality of variables.

To allow comparability with studies employing different methodologies, ROC curves for the algorithms studied were established at 42, 90, and 365 days of survival, matching the survival prediction thresholds of the SORG ML method. This could not be done for the OPTImodel method as it does not employ a quantitative scale. Following bibliographic recommendations, AUCs above 0.8 were deemed optimal, while those below 0.7 were considered insufficient. Statistical analysis was performed using SPSS version 22.0.

## 3 Results

Of the 622 patients identified with vertebral metastases at the time of diagnosis, 49 were excluded for not meeting inclusion criteria. Specifically, 12 were excluded due to the unavailability of diagnostic imaging for analysis, and 37 due to missing data in the collected variables. The final number of patients included in the study was 573.

The mean age at diagnosis was 69.1 years (SD = 11.4). Of the patients, 361 (63%) were male, with a mean BMI of 27.1 (SD = 5.33), and 362 (63.2%) had comorbidities as assessed by the Charlson Comorbidity Index.

In terms of functional status, 287 patients (50%) had a Karnofsky score of 70, indicating good functional condition, while 143 (25%) had an initial score of 60 or less (median = 70, Q1 = 60, Q3 = 80). A total of 516 patients (90.1%) had no spinal cord involvement according to the ASIA scale. Table 1 presents the analytical values of the patients at the time of diagnosis.

**TABLE 1:** Analytical values at diagnosis.

Parameter	Median (Q1–Q3)
Hemoglobin (g/dL)	11.9 (10.6–13.5)
Platelets (×10^3^/μL)	241 (188–314)
Leukocytes (×10^3^/μL)	7.98 (6.01–10.5)
Lymphocytes (×10^3^/μL)	1.44 (0.950–2.02)
Neutrophils (×10^3^/μL)	5.22 (3.75–7.94)
Creatinine (mg/dL)	0.88 (0.7–1.12)
INR (International Normalized Ratio)	1.11 (1.04–1.19)
Alkaline phosphatase (IU/L)	141 (93–234)
Albumin (g/L)	34 (32–35.6)

The mean number of vertebral metastases at diagnosis was 2.89 (SD = 1.6), with a mean of 3.93 (SD = 1.88) total bone metastases per patient at diagnosis. A total of 272 patients (47.5%) had visceral metastases, of which 160 (27.9%) were considered potentially treatable with curative or disease‐modifying intent using any of the three standard treatment modalities (chemotherapy or targeted therapy, radiotherapy, or surgery), as opposed to purely palliative treatment aimed at symptom control only. Additionally, 305 (53.3%) had lymphatic metastases, and 29 (5.1%) had brain metastases. Of the total sample, 342 patients (56.6%) had received prior systemic therapy as curative intent, either for the primary tumor or its metastases.

The survival percentage at the end of the 10‐year study interval was 8.63%. The most frequent primary tumor was lung cancer, with 147 cases (25.6%), including both small cell and non‐small cell subtypes, followed by prostate cancer with 146 cases (25.3%) and breast cancer with 118 cases (20.14%). Table 2 shows the frequencies of the other histological diagnoses. The highest survival rates were observed in prostate and breast cancers, while the lowest were found in bladder, pancreatic, and colorectal cancers.

**TABLE 2:** Most common histologies.

Histology	*n*	%
Lung (all subtypes)	147	25.6
Prostate (all subtypes)	145	25.3
Breast (all subtypes)	115	20.14
Non‐small cell lung	103	17.9
Hormone‐dependent breast	74	13
Small cell lung	44	7.7
Hormone‐independent breast	41	7.14
Others	40	7
Colorectal	29	5.1
Kidney	29	5.1
Bladder	17	3
Unknown	16	2.8
Liver	9	1.6
Pancreas	9	1.6
Larynx	6	1.1
Thyroid	5	0.9

Regarding the models, the Tokuhashi algorithm divides survival into three categories: low (< 6 months; 0–8 points), intermediate (6–12 months; 9–11 points), and high (> 1 year; 12–15 points) [9]. In our study, 252 patients (44.11%) were classified in the low survival group, of which 188 (74.9%) were correctly categorized as alive or deceased. In the intermediate survival group (9–11 points), which included 199 patients (34.8%), only 30 (15.2%) were correctly categorized. In the high survival group (12–15 points), which included 122 patients (21.09%), 93 (77.5%) were correctly categorized. Sensitivity was 0.67 for the low survival group, 0.42 for the intermediate group, and 0.43 for the high group, with specificities of 0.78, 0.66, and 0.92, respectively. Positive predictive values were 0.75 for the low survival group, 0.152 for the intermediate group, and 0.73 for the high survival group. Negative predictive values were 0.7, 0.89, and 0.73, respectively. The overall accuracy of the Tokuhashi algorithm was 0.5682 (95% CI: 0.5063–0.5895).

**FIGURE 1:**
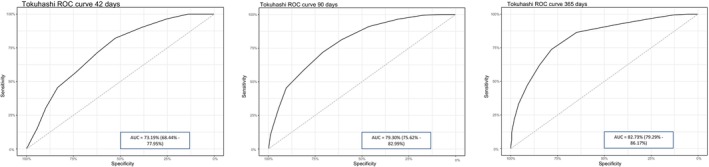
ROC curves for Tokuhashi at 42 (left), 90 (middle), and 365 days (right).

In our study, the Tokuhashi method demonstrated adequate predictive capacity for survival below 6 months and above 1 year, but its predictive ability declined significantly for intermediate survival values. As shown in Figure 1, the ROC curves provided AUCs of 73.19% (95% CI: 68.44–77.95) for survival at 42 days, 79.3% (95% CI: 75.62–82.99) for 90 days, and 82.73% (95% CI: 79.29–86.17) for 365 days.

The Tomita algorithm considers four categories: 2–4 points (more than 2 years), 4–6 points (1–2 years), 6–8 points (6–12 months), and 8–10 points (< 3 months) [11]. In our study, 201 patients (35.2%) were in the 2–4 points group, of which 87 (43.3%) were correctly categorized as alive or deceased. In the 4–6 points group, which included 143 patients (25%), only 22 (15.4%) were correctly categorized. In the 6–8 points group, with 99 patients (17.3%), 29 (29.3%) were correctly categorized, while in the 8–10 points group, with 128 patients (22.4%), 91 (71.1%) were correctly categorized. Sensitivity for the Tomita method was 0.68 for the 2–4 points group, 0.25 for the 4–6 points group, 0.19 for the 6–8 points group, and 0.45 for the 8–10 points group, with specificities of 0.74, 0.75, 0.83, and 0.9, respectively. Positive predictive values were 0.43, 0.15, 0.29, and 0.71, and negative predictive values were 0.9, 0.84, 0.74, and 0.75, respectively. The overall accuracy of the algorithm was 0.4011 (95% CI: 0.3606–0.4426).

**FIGURE 2:**
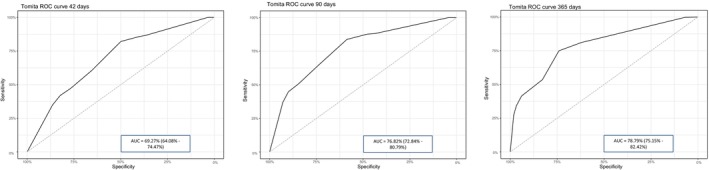
ROC curves for Tomita at 42 (left), 90 (middle), and 365 days (right).

Similar to Tokuhashi, the Tomita algorithm showed high predictive capacity for extreme survival values (< 3 months) but lower accuracy for survival over 2 years, with prediction success rates below 30% for intermediate categories. For the Tomita method, the AUCs on the ROC curves were 69.27% (95% CI: 64.08–74.47) for 42 days, 76.82% (95% CI: 72.84–80.79) for 90 days, and 78.79% (95% CI: 75.15–82.42) for 365 days (Figure 2).

**FIGURE 3:**
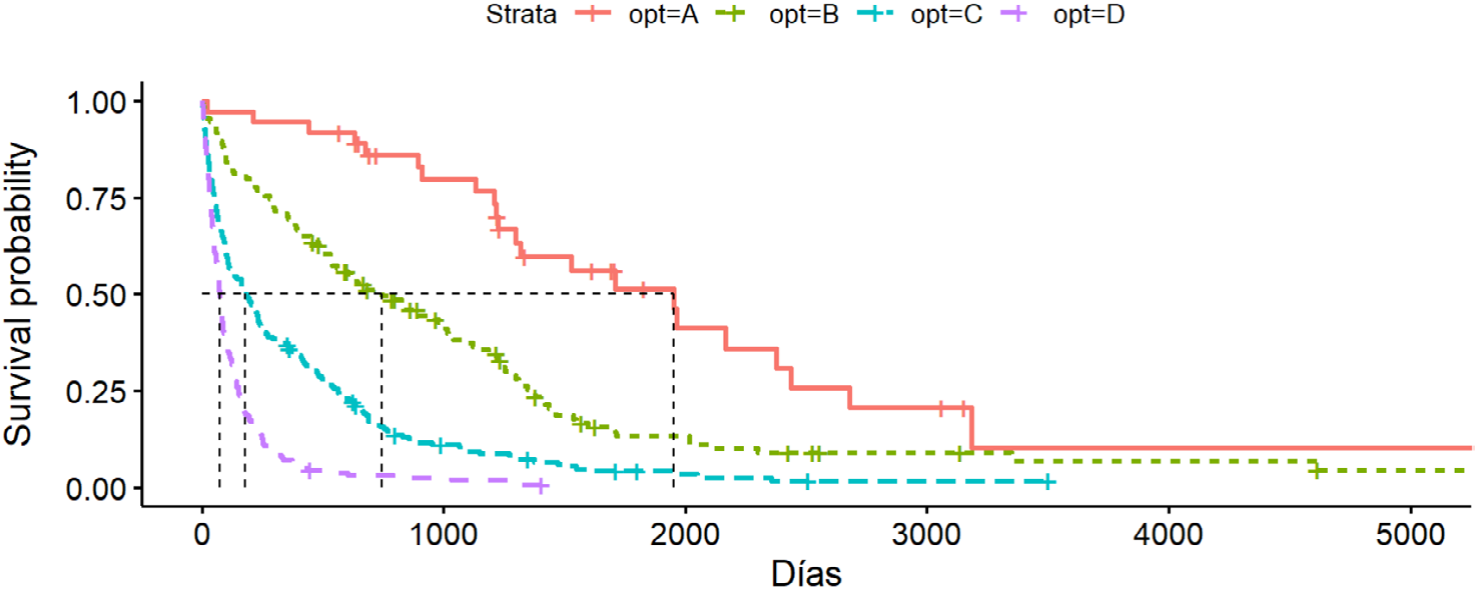
Kaplan–Meier curve for each interval in OPTImodel.

For OPTImodel, four categories are defined: D (< 90 days), C (90–180 days), B (180–365 days), and A (more than 365 days) [17]. Figure 3 shows the Kaplan–Meier curve for each OPTImodel interval. In our study, 36 patients (6.28%) were in group A, of which 34 (94.4%) were correctly categorized as alive or deceased; 134 patients (23.4%) were in group B, with 16 (11.9%) correctly categorized; 213 patients (37.2%) were in group C, with 31 (14.6%) correctly categorized; and 185 patients (32.1%) were in group D, with 110 (59.5%) correctly categorized.

The sensitivity values for the groups were 0.16 for group A, 0.22 for group B, 0.38 for group C, and 0.54 for group D. Specificity values were 0.99, 0.76, 0.63, and 0.8, respectively. Positive predictive values were 0.95 for group A, 0.12 for group B, 0.14 for group C, and 0.59 for group D, with corresponding negative predictive values of 0.66, 0.87, 0.86, and 0.76. The overall accuracy of the OPTImodel algorithm was 0.3368 (95% CI: 0.2981–0.3773).

As observed with other methods, OPTImodel showed high prediction success for extreme survival values (> 1 year or < 90 days) but poor predictive success rates (below 15%) for intermediate survival values. Due to the study's methodology, ROC curves could not be obtained for OPTImodel as it does not use a quantitative scale.

The SORG ML method does not group patients by prognosis but directly calculates the probability of survival at 42, 90, and 365 days [21]. Due to this difference from other methods, calculations were made directly for each survival threshold.

For the 42‐day survival threshold, sensitivity was 0.21, specificity was 0.8, the positive predictive value was 0.8, and the negative predictive value was 0.21. The accuracy of the SORG ML algorithm for the 42‐day threshold was 0.3333 (95% CI: 0.2947–0.3737). For the 90‐day threshold, sensitivity, specificity, positive predictive value, and negative predictive value were 0.75, 0.20, 0.63, and 0.31, respectively. The accuracy for this threshold was 0.5561 (95% CI: 0.5143–0.5974). For the 365‐day threshold, sensitivity, specificity, positive predictive value, and negative predictive value were 0.24, 0.8, 0.42, and 0.63, respectively. The accuracy of the SORG ML algorithm for the 365‐day threshold was 0.3333 (95% CI: 0.2947–0.3737).

**FIGURE 4:**
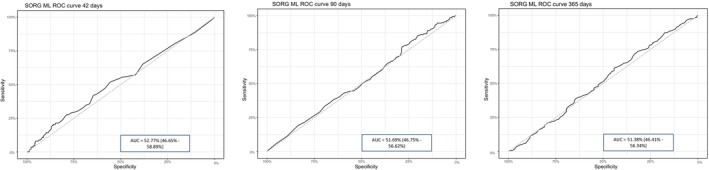
ROC curves for SORG ML at 42 (left), 90 (middle), and 365 days (right).

For the 42‐day threshold, the AUC on the ROC curve was 52.77% (95% CI: 46.65–58.89); for the 90‐day threshold, it was 51.69% (95% CI: 46.75–56.62); and for the 365‐day threshold, it was 51.38% (95% CI: 46.41–56.34). Figure 4 shows the ROC curves for SORG ML at 42 (A), 90 (B), and 365 days (C).

The method with the highest accuracy was SORG ML at 365 days (0.5877), followed by SORG ML at 90 days (0.5561) and Tokuhashi (0.5482). Table 3 summarizes the accuracy and AUC of the prognostic algorithms, ranking them from highest to lowest accuracy and showing the AUC for each method. Only the Tokuhashi method achieved AUC values > 70% across all intervals, while Tomita yielded results close to this threshold, and SORG ML showed significantly lower values.

**TABLE 3:** Summary of prognostic algorithm accuracy and AUC.

Algorithm	Accuracy
Tokuhashi	0.5482
OPTIModel	0.3368
Tomita	0.4011
SORG 42 days	0.3333
SORG 90 days	0.5561
SORG 365 days	0.5877

Algorithm	AUC
Tokuhashi 42 days	73.19%
Tokuhashi 90 days	79.3%
Tokuhashi 365 days	82.73%
Tomita 42 days	69.27%
Tomita 90 days	76.82%
Tomita 365 days	78.79%
SORG ML 42 days	52.77%
SORG ML 90 days	51.69%
SORG ML 365 days	51.38%

## 4 Discussion

The predictive accuracy of the studied methods contrasts, particularly in the case of the SORG ML algorithm, with the numerous studies supporting its use. These studies mainly involve external validations [22, 23, 24] and comparative analyses [6, 25]. However, our results find support in studies such as those by Truong et al. [26] or Hibberd et al. [27] Regarding the Tokuhashi method, external validation attempts, like the one conducted by Hernandez‐Fernandez et al. [28], have shown results consistent with ours.

We conducted a literature review that identified 45 relevant articles from which we extracted the AUC values of the algorithms at 3 months, 6 months, and 1 year, as these are the most frequently reported intervals. We also recorded the overall conclusions and whether the sample included only surgically treated patients for vertebral metastases or, as in our study, made no distinction between treated and untreated patients. The average AUCs from the review were as follows: 0.66, 0.63, and 0.67 for Tokuhashi at 1, 3, and 12 months, respectively; 0.61, 0.63, and 0.69 for Tomita; 0.73, 0.67, and 0.75 for SORG ML; and 0.67 overall for OPTImodel.

The data from our study align with published results for the Tomita and Tokuhashi methods but not for SORG ML, where our AUC values were barely above random chance (0.51–0.52), as shown in Table 4, which lists the average AUCs from the reviewed studies. Only two studies applied any variant of the SORG method to a sample that did not distinguish between operated and non‐operated patients [26, 29]. In the study by Tarabay et al. [30], the AUC decreased significantly (0.59 at 1 year), while in Su et al. [31], the AUC remained at 0.78. The other 17 studies involving SORG ML used samples exclusively comprising surgically treated patients [6, 17, 18, 19, 22, 23, 24, 25, 29, 32, 33, 34, 35, 36, 37, 38].

**TABLE 4:** Mean AUC in the literature review.

Metric	Tokuhasi 3 m	Tokuhasi 6 m	Tokuhasi 1a	Tomita 3 m	Tomita 6 m	Tomita 1y	SORG 3 m	SORG 6 m	SORG 1y	OPTImodel
Overall mean	0.66	0.63	0.69	0.61	0.63	0.69	0.73	0.67	0.75	0.67
Non‐distinction mean	0.66	0.63	0.64	0.62	0.64	0.63	0.72	0.67	0.69	0.64
Operated mean	0.66	0.63	0.68	0.6	0.61	0.66	0.75	0.7	0.78	0.73
Our study	0.73	0.79	0.82	0.69	0.76	0.78	0.52	0.51	0.51	NA

Abbreviations: m, months; NA, not applicable; y, year.

The discrepancy between our results and those of other authors for the SORG ML method could be partially explained by differences in the patient populations studied, as our sample included both operated and non‐operated patients without distinction.

Moreover, biases may exist in validating prognostic algorithms in studies that include only surgically treated patients, as this subgroup is subjectively more favorable. Consequently, the majority of patients diagnosed with vertebral metastases who do not undergo surgery are excluded from such analyses.

The main limitations of this study relate to the fact that our patients were older and had more comorbidities compared to those in other studies [23]. Small variations were also noted in the epidemiology of primary tumors, with a higher proportion of prostate and colorectal cancers in our sample, potentially attributable to the older age of the patients or geographic variations.

Our sample was broad and heterogeneous, covering a 10‐year study period, which may have introduced differences in the prognosis of older versus more recent patients. Additionally, quantitative comparisons with the OPTImodel method could not be performed due to the lack of AUC data. We did not record which patients underwent surgery, which could provide additional information on prognostic subcategories.

This research group is currently conducting a study comparing outcomes in surgically and non‐surgically treated patients with vertebral metastases. Multidisciplinary oncological committee decisions were also not recorded, as these were often absent from the retrospective review of clinical records for many patients in the study. We believe this could be a relevant factor for guiding prognosis.

## 5 Conclusions

Overall, the methods compared in our study demonstrated relatively low predictive accuracy. In our patient sample, the OPTImodel and SORG ML methods failed to outperform the classic Tomita and Tokuhashi methods in predicting survival in patients with vertebral metastases. Our findings align with those of other authors regarding the Tokuhashi and Tomita methods but not for SORG ML.

It seems, therefore, that none of the four methods studied possesses sufficient prognostic quality for generalization in clinical practice, which may significantly limit their utility. Nonetheless, individual clinical parameters such as general health status, disease extent (e.g., visceral metastases, multiple bone metastases), and analytical values remain fundamental factors in evaluating these patients.

## Author Contributions

**Julián Cabria Fernández:** conceptualization, investigation, methodology, writing – original draft, writing – review and editing. **Pablo González‐Herráez Fernández:** investigation, methodology, software. **Javier Mateo Negreira:** validation, visualization, data curation, supervision, resources. **Pedro Arcos González:** formal analysis, project administration, supervision, resources, data curation, writing – review and editing.

## Funding

The authors have nothing to report.

## Ethics Statement

This study was approved by the Research Ethics Committee of the Principality of Asturias (file number CEImPA 2024.307). According to regional regulations and Spanish legislation (Biomedical Research Law 14/2007), the study was considered exempt from requiring informed consent due to its retrospective, observational, and anonymized nature, in which no personally identifiable patient data were collected.

## Conflicts of Interest

The authors declare no conflicts of interest.

## Data Availability

The data that support the findings of this study are available from the corresponding author upon reasonable request.
